# P-1935. Persistent Candidemia: Predictors and Outcomes in a Multicenter Matched Analysis

**DOI:** 10.1093/ofid/ofaf695.2103

**Published:** 2026-01-11

**Authors:** David Bayless, Jack W McHugh, Omar M Abu Saleh, Paschalis Vergidis, Douglas Challener

**Affiliations:** Mayo Clinic, Rochester, MN; Mayo Clinic, Rochester, MN; Mayo Clinic, Rochester, MN; Mayo Clinic, Rochester, MN; Mayo Clinic, Rochester, MN

## Abstract

**Background:**

Persistent candidemia (PC) occurs in a minority of *Candida* bloodstream infections (BSIs) but is associated with increased morbidity. Comparative data on patient profile, species distribution and outcomes are limited.

**Table 1:**
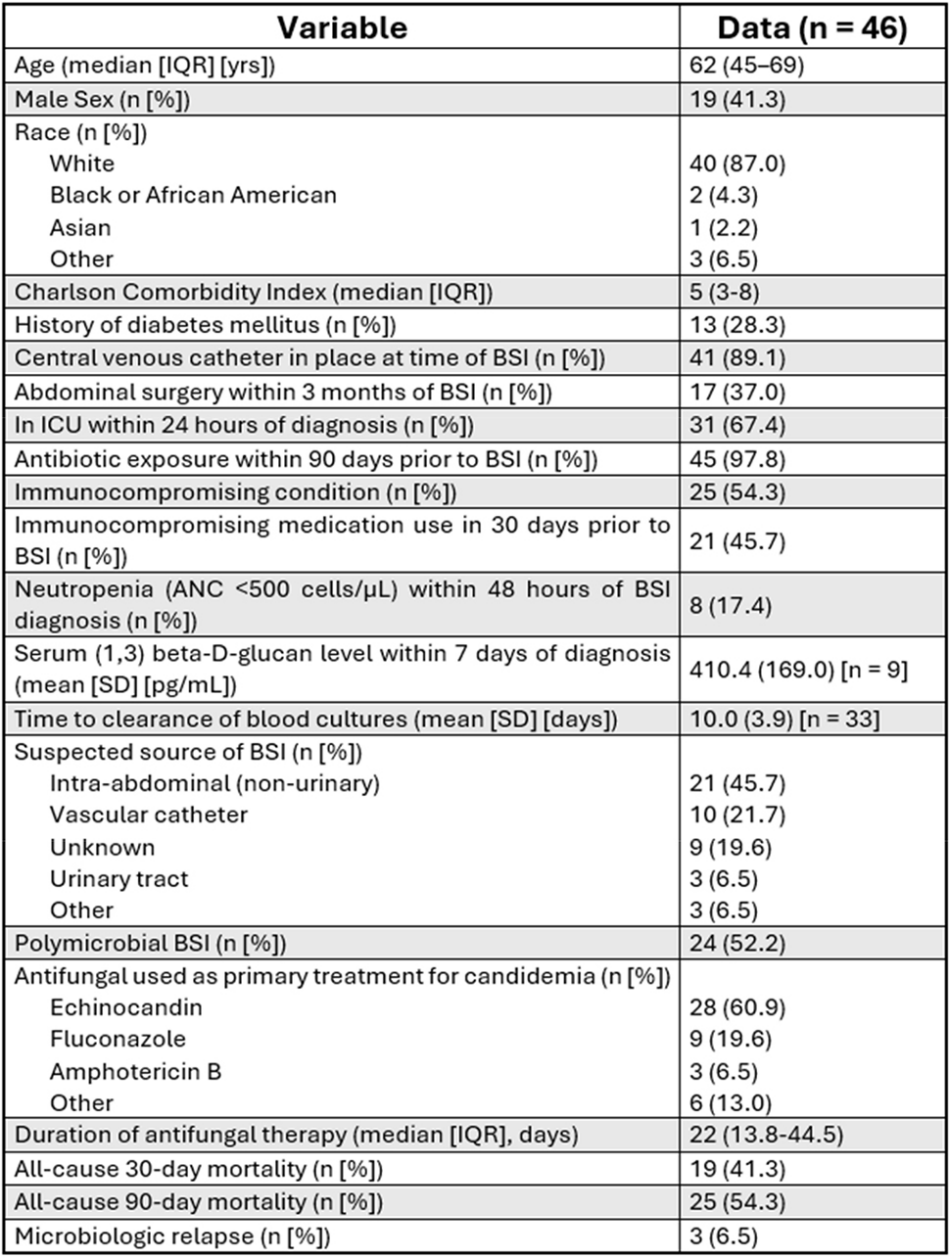
Clinical profile and outcomes of patients with persistent candidemia in the primary study cohort. ANC = absolute neutrophil count. SD = standard deviation.

**Table 2:**
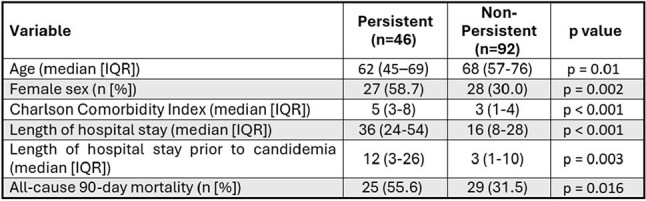
Comparative analysis of persistent candidemia and non-persistent candidemia cases.

**Methods:**

All candidemia episodes at three academic medical centers (2018-2025) were reviewed. Persistence was defined as ≥72 hours of positive blood cultures after appropriate antifungal initiation. Each PC case was matched 1:2 to non-PC controls who survived ≥72 hours. Demographics, Charlson Comorbidity Index (CCI), *Candida* species distribution, and outcomes were used for comparative analysis. Continuous variables were summarized as median (interquartile range [IQR]) and compared with Wilcoxon rank-sum, while categorical data (n%) were analyzed by Fisher’s exact test (species by χ²), yielding odds ratios (ORs) with 95% confidence intervals (CIs). Two-tailed p < 0.05 denoted significance.

**Figure:**
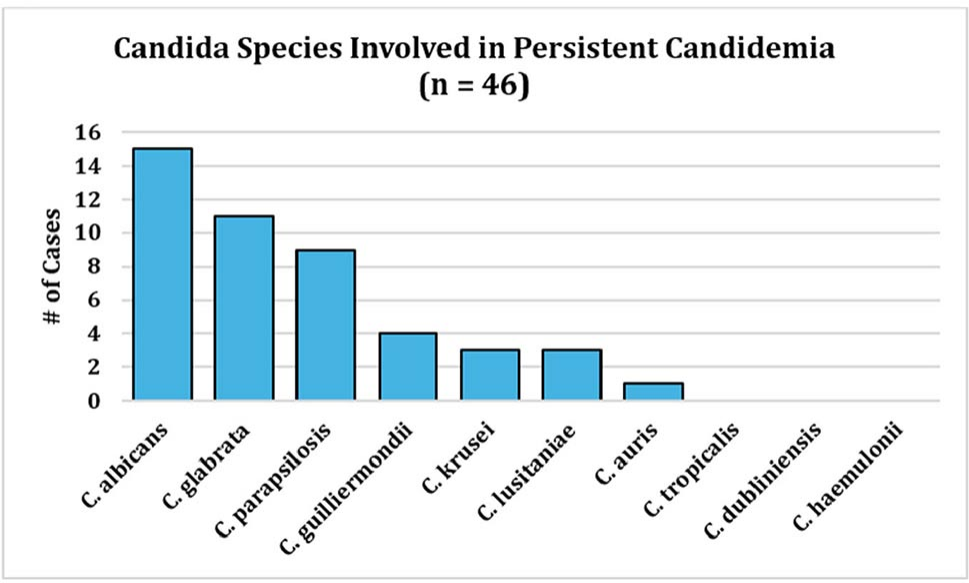

**Figure:**
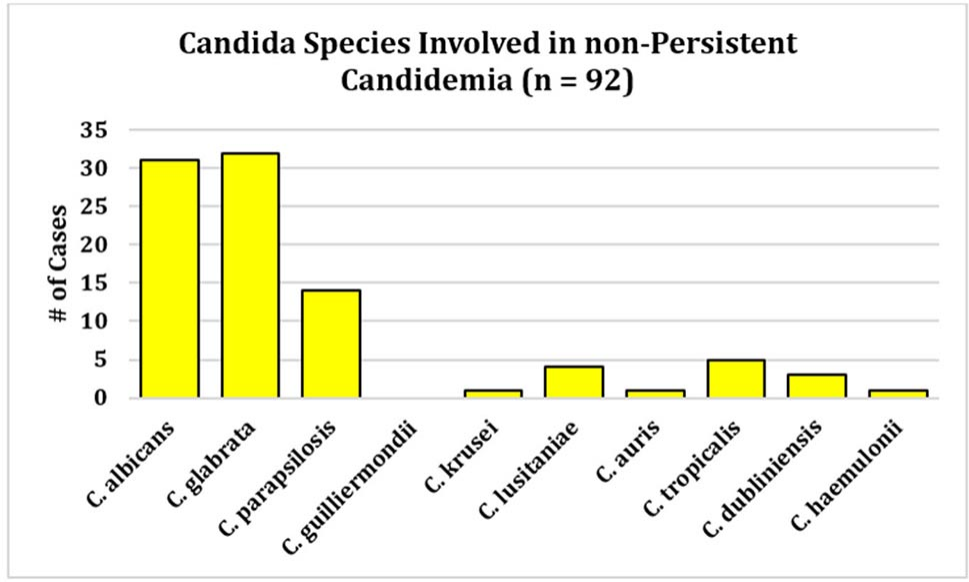

**Results:**

Each PC case (n = 46) was matched 1:2 to non-PC controls (n = 92). Clinical profiles and outcomes of PC cases are presented in Table 1. Most PC patients (54.3%) had an immunocompromising condition, and non-urinary intra-abdominal sources were the most frequently suspected infection source (21/46; 45.7%). All but one of 46 PC cases had antibiotic exposure within the preceding 90 days. Comparative demographics and outcomes for PC cases and non-PC controls are displayed in Table 2. PC patients were younger (median 62 yr, IQR 45–69 vs 68 yr, 57–76; p = 0.01), more often female (59% vs 30%; OR 3.3, 95% CI 1.5–7.1; p = 0.002), and had higher comorbidity (CCI 5, IQR 3–8 vs 3, 1–4; p < 0.001). Species distribution differed (χ² p = 0.04), driven by *C. guilliermondii* over-representation in PC cases (9% vs 0%) (Figures 1 and 2). Hospital stay was longer in PC cases (median 36 d, 24–54 vs 16 d, 8–28; p < 0.001) and time from admission to first culture greater (12 d, 3–26 vs 3 d, 1–10; p = 0.003). 90-day mortality was 54.3% (25/46) in PC cases versus 31.5% (29/92) in controls (OR 2.57, 95% CI 1.17–5.71; p = 0.016).

**Conclusion:**

In this 7-year multicenter study, there were significant differences in demographics, microbiology, and clinical outcomes between PC and non-PC cases, including higher short-term mortality rates with PC in univariate analysis.

**Disclosures:**

Paschalis Vergidis, MD, MSc, Current Fungal Infection Reports: Honoraria|F2G: Grant/Research Support|Merck Manuals: Honoraria|Mundipharma: Grant/Research Support|Scynexis: Advisor/Consultant|Scynexis: Grant/Research Support

